# Vaccine Virus Victorious

**Published:** 1885-12

**Authors:** 


### VACCINE VIRUS VICTORIOUS.

The good and godly people who dwell in the classic precincts of Fairmount, and whose devotions, or other duties, called them up early last Sunday morning, were treated to the novel Sabbath sight of a sprint-race, or go-as-you-please contest, between a pumpkin-pie-colored cow and the vacher whose ranch is at the foot of Fairmount, which, while it lasted, was full of excitement to the contestants and not uninteresting to the outsiders.

It is a good cow and a virtuous, but, away back among her ancestry there was dissipation, debauchery and other forms of badness, which occasionally, comes out in streaks in the present generation. With periodic regularity this otherwise exemplary bovid has times of wanting to stay out all night, and raise sheol with the neighbor's gardens, and get on a beautiful booze by eating inordinate quantities of winter apples. Last Saturday night she was on a regular tear, and evidently determined that she wouldn't go home until morning. Well, the morning came and she didn't, but the old agronomic heard of her whereabouts, and started out for her with sorrow in his heart and a savage looking club in his hand.

They met by chance, the usual way, but when the old farmer would fain have persuaded her to seek the solitude of her vachery, there to put her head a-soak, and otherwise do penance for the night's debauchery, she mooed him to scorn, and elevating her spinaker boomlet, she set sail for Southboro.

Then ensued a fair test of the speed capacity of four legs or two. It was neck and nose for a long time, but when the distance post was reached, two legs had the best of it by half a neck, and both contestants were going beautifully. But at this point it occurred to the two legged trotter that the proper thing to do was to take the pole from his four legged adversary, and shut her out, so to speak, and this he essayed to do. But the party of the other part seeing the end he had in view, hit it, and thereby caused a catastrophe.

George Stephenson said, in answer to a question as to what would happen if a cow should get on the track of a locomotive going at full speed, that "it would be bad for the coo." When an irresistible force strikes an immovable ponderosity there is a smash. This is not theory with us; we know whereof we affirm. In this particular case it was the steam engine that was derailed, and the cow kept the track.

When the dust of the conflict had cleared away, a solitary herdsman might have been seen slowly ascending the grassy bank by the roadside, where he sat himself down to reflect on the mutability and unreliability of all mundane things. Had any of the neighbors been near enough to listen to his soliloquy, they might have thought he was repeating his Sunday school lesson, but he wasn't. He was just thinking with poor old King Lear

How sharper than a serpent's tail it is
To have a toothless cow.

Especi lly one given to Saturday night debauchery and Sunday morning sheolness.

When he got some of the dirt out of his eyes and mouth, he took a look round for the cause of his downfall. There she stood, not thirty feet away.

In vaccine meditation fancy free, with a smile of derision on her face, and a big apple in her mouth.

Then a small boy came along and taking pity on the sorrows of the poor old man, drove the cow to the historic barn at the foot of Fairmount, and tied her up in her thousand dollar stall, where she will stay until the butcher comes to take her where all good cows, and bad, must finally meat.—*The Marlboro Times.*

